# Awareness of hereditary breast cancer and readiness for genetic testing among newly diagnosed patients with breast cancer in Nigeria: a multicentre study

**DOI:** 10.1186/s13053-026-00349-8

**Published:** 2026-07-27

**Authors:** Funmilola O. Wuraola, Jenine Ramruthan, Victoria L. Mango, Nneka Sunday-Nweke, Olayide Agodirin, Anna Dare, Adewale A. Aderounmu, Mary Ogunyemi, Boladuro Olawale, Saheed Lawal, Anna T. Santiago, Olalekan Olasehinde, Adewale O. Adisa, Emma Reel, Olusegun I. Alatise, Tulin D. Cil

**Affiliations:** 1 Department of Surgery, Faculty of Clinical Sciences, Obafemi Awolowo University, Ile-Ife, Nigeria; 2 African Research Group for Oncology, Obafemi Awolowo University Teaching Hospitals Complex, Ile-Ife, Nigeria; 3 Department of General Surgery, Princess Margaret Cancer Centre - University Health Network, 700 University Avenue, Toronto, Canada; 4 Department of Radiology, Memorial Sloan Kettering Cancer Center, New York, NY USA; 5 Department of Surgery, Alex Ekwueme Federal University Teaching Hospital, Abakaliki, Nigeria; 6 Department of Surgery, University of Ilorin, Ilorin, Nigeria; 7 Department of Surgery, University of Toronto, Toronto, ON Canada; 8 Department of Surgery, Dalla Lana School of Public Health, Toronto, ON Canada; 9 Department of Biostatistics, University Health Network, Toronto, ON Canada

**Keywords:** Hereditary breast cancer, *BRCA1/2*, Genetic testing, Patient knowledge, Nigeria

## Abstract

**Background:**

Germline *BRCA1/2* pathogenic variants account for up to 11% of breast cancer (BC) cases in Nigeria, approximately twice the prevalence reported in high-income populations. However, access to hereditary cancer services remains limited and patient awareness is largely uncharacterized. Prior studies have assessed knowledge of genetic testing among healthy populations or following structured genetic counselling, but none have evaluated baseline awareness at the point of clinical diagnosis. This multicentre study measured baseline awareness of hereditary BC and willingness to undergo *BRCA1/2* testing among patients newly diagnosed with BC at three tertiary hospitals, as a pre-implementation evaluation for hereditary cancer services in this setting.

**Methods:**

We conducted a cross-sectional, multicentre study of 230 newly diagnosed patients with histologically confirmed invasive BC recruited between March 2022 and October 2023 at three tertiary teaching hospitals in Nigeria (Osun, Kwara, and Ebonyi States, representing the South West, North Central, and South East geopolitical zones). A structured, interviewer-administered questionnaire was used to assess knowledge of BC risk factors and symptoms, recognition of hereditary BC, awareness of *BRCA1/2*, and willingness to undergo germline testing and engage in family disclosure. Factors associated with knowledge and willingness were examined via univariable and multivariable regression.

**Results:**

The median age was 47 years; 66.5% were of low socioeconomic status, and 33.0% had a tertiary education. Most patients presented with advanced disease (stage III/IV: 73.0%), and among those with available data, 76.3% had triple-negative BC. Recognition that BC can be hereditary was limited (55.6%, 95% CI 49.1–61.9%), and prior awareness of *BRCA1/2* was extremely low (4.8%; 95% CI 2.7–8.4%). According to the multivariable analysis, recognition of hereditary BC was independently associated with education (*p* < 0.001) and hospital centre (*p* = 0.024). Despite limited genetic literacy, willingness to undergo *BRCA1/2* genetic testing was high (95.7%), with most participants willing to inform siblings (94.8%) and encourage genetic testing for family members (96.1%).

**Conclusions:**

Among newly diagnosed patients with BC across three Nigerian tertiary centres, awareness of hereditary BC and *BRCA1/2* was low, yet willingness to undergo genetic testing was near-universal. Together with previously reported provider readiness, these findings point to service availability rather than patient or provider attitudes as the principal barrier to introducing hereditary BC services in Nigeria. As these services are being developed in Nigeria and comparable settings across sub-Saharan Africa, the corresponding priority will be to design consent pathways, patient-facing education materials, and provider training that addresses the gap between patient willingness and genetic literacy.

**Supplementary Information:**

The online version contains supplementary material available at https://doi.org/10.1186/s13053-026-00349-8.

## Introduction

Breast cancer is the most common malignancy and leading cause of cancer mortality among women in Nigeria [1,2], with an age-standardized incidence of 54.3 per 100,000 that has risen steadily over the past two decades, consistent with the broader cancer transition across sub-Saharan Africa (SSA) [1, 3]. More than 80% of Nigerian patients are diagnosed at stage III or IV, at younger ages, and with a disproportionately high prevalence of high-grade, triple-negative tumors, compared with patients in North America and Europe, where early-stage diagnosis and treatment are more common [4–6].

A substantial minority of this disease burden is hereditary. Pathogenic variants (PVs) in *BRCA1* and *BRCA2* (BReast CAncer genes 1 and 2) account for most hereditary breast cancers, with additional contributions from other high risk genes such as *PALB2* (Partner and Localizer of *BRCA2*) and *TP53* (Tumour protein p53) [7]. Carriers of *BRCA1/2* PVs face substantially elevated lifetime cancer risks: up to 70% of carriers will develop breast cancer, compared with 13% of women in the general population [7, 8]. The prevalence of *BRCA1/2* PVs varies by population and remains poorly characterized outside of high-income populations [9, 10]. Globally, *BRCA1/2* PVs account for an estimated 5–10% of all breast cancers [11]. However, emerging data from Nigeria suggest a comparatively greater burden, with more than 11% of patients with breast cancer harboring *BRCA1/2* PVs; when other high/moderate penetrance genes such as *PALB2* and *TP53* are included, as many as 13% of cases may be attributable to inherited susceptibility in this population [12].

This high hereditary prevalence, combined with younger age at diagnosis and high rates of triple-negative disease, underscores the potential clinical relevance of hereditary cancer risk assessment in Nigeria. However, Nigeria and many countries in SSA lack national frameworks for hereditary cancer services and access to cancer genetic testing and counselling services is only available through a few private laboratories or research driven projects [13, 14]. Consequently, awareness and understanding of hereditary breast cancer and genetic testing remain low even among healthcare providers (HCPs) [13].

A greater understanding of hereditary breast cancer risk in Nigeria is needed. However, this must occur alongside efforts to understand patient perceptions and acceptability of genetic testing [15]. Available evidence from Nigeria suggests that despite low genetic literacy, women demonstrate high receptivity toward genetic testing. A survey of professional women (university lecturers and bankers) found that 84.7% had limited knowledge of breast cancer genetics and that only 13.6% were aware of *BRCA1/2* genes; yet 87.3% reported willingness to undergo germline genetic testing where available [15]. Similarly, clinic-based studies among patients with cancer in South West Nigeria demonstrated high acceptability following structured pre-test genetic counselling, with 86.6% willing to undergo testing and 71.1% willing to pay out of pocket, though willingness to pay was highly price-sensitive [16]. Taken together, this evidence demonstrates high acceptability of germline testing following structured pre-test genetic counselling, establishing that willingness is not a barrier once patients are appropriately informed. However, in most Nigerian centres such counselling infrastructure does not yet exist, and genetic testing will first be introduced to patients at the point of clinical diagnosis. Quantifying baseline willingness and awareness at this earlier stage, is therefore necessary to characterize the conditions under which hereditary cancer services will be implemented in practice, and to identify the specific educational and counselling components that must be developed to support informed decision-making.

The present study aims to evaluate baseline awareness of hereditary breast cancer and willingness to undergo *BRCA1/2* genetic testing at the point of diagnosis across multiple centres, representing three geopolitical zones and serving populations that include the country’s largest ethnic groups (Yoruba, Hausa and Igbo).

## Methods

### Study setting

We conducted a multicentre, cross-sectional study at three tertiary teaching hospitals across three geopolitical zones in Nigeria: (1) Alex Ekwueme Federal University Teaching Hospital (FETHA), Abakaliki, Ebonyi State; (2) University of Ilorin Teaching Hospital (UITH), Ilorin, Kwara State; and (3) Obafemi Awolowo University Teaching Hospitals Complex (OAUTHC), Ile-Ife, Osun State. All three sites were similar in infrastructure and clinical capacity but served populations from distinct geopolitical zones and predominantly different ethnic groups (Igbo, Hausa and Yoruba respectively). Patients ≥ 18 years of age presenting to OAUTHC, UITH, and FETHA with a histologically confirmed diagnosis of invasive breast cancer were eligible for inclusion in the study, regardless of their family history. The study was conducted in accordance with the Declaration of Helsinki. Ethical approval was obtained from the institutional review boards of all participating centers (#OAUTHC ERC/2022/03/03, #UITH ERC PIN/2022/03/1086 and #AEFUTHA REC/VOL.3/2022/048). Written informed consent was obtained from all individual participants included in this study prior to enrolment. Consent procedures and study materials were available in English and in the participants’ preferred local language (Yoruba, Igbo, or Hausa).

## Data collection

All study information, consent forms, questionnaires and study instructions (written and verbal) were offered in English or the local language, based on the participant’s preference. Participants completed a baseline questionnaire, which included demographic information (age, ethnicity, education level, socioeconomic status). Medical history (including tumor size, location, TNM staging [AJCC 8th edition], histopathological diagnosis, tumor grade, and hormone receptor status based on immunohistochemistry) was prospectively collected at the time of enrollment in the study or as soon as the relevant information became available, by the study research assistant at each site.

### Questionnaire development and administration

A structured, interviewer-administered 49-item questionnaire developed for this study was used to assess participants’ baseline knowledge across four domains: (1) knowledge of breast cancer risk factors (19 items), (2) knowledge of breast cancer symptoms (16 items), (3) knowledge of hereditary breast cancer (9 items); and (4) perceptions and willingness regarding genetic testing (5 items). Domains 1–3 used yes/no/did not answer responses and domain 4 was open-ended. No Likert-type scales were included. The correct-response key for domains 1–3 was defined a priori by the study team. The questionnaire was translated from English into the three predominant local languages (Yoruba, Igbo, and Hausa) by native-speaking members of the clinical research team, and then independently back-translated to verify fidelity. Trained interviewers administered the questionnaires in the participants’ preferred language to ensure comprehension and accuracy.

### Sample size

A target enrollment of 230 patients was established based on estimated annual breast cancer case volumes across the three participating centres [4].

### Statistical analysis

Data were collected and managed using REDCap electronic data capture tools hosted at OAUTHC. Descriptive statistics were used to summarize sociodemographic, clinical, and knowledge-related variables. Patient responses to the questionnaires were reported as frequencies and percentages for categorical options or means, medians, standard deviations, and ranges for numeric responses. Knowledge items were summarized according to actual patient responses and correct/incorrect responses according to the answer key. Distributions of knowledge scores and item-level responses were additionally visualized using bar charts and box plots. The Wilcoxon rank-sum or Kruskal-Wallis test and Chi-squared test were used to determine patient factors associated with knowledge and willingness to conduct genetic testing. Statistical analysis was performed using R version 4.5.1 (R Core Team, 2025).

## Results

### Participant characteristics

Between March 2022 and October 2023, 230 patients with histologically confirmed invasive breast cancer were enrolled across three sites: 43.0% (99/230) were recruited from OAUTHC, 32.2% (74/230) from UITH, and 24.8% (57/230) from FETHA. Nearly all participants were female (99.1%, 228/230). The median age of the cohort was 47 years (range: 23–87 years). Most participants were Yoruba (71.3%, 164/230), followed by Igbo (23.5%, 54/230), and Hausa (0.9%, 2/230). Two-thirds were of low socioeconomic status (SES) (66.5%, 153/230), with 30.9% (71/230) classified as middle income, and only 2.2% (5/230) was high income. With respect to educational background, 33.0% (76/230) had tertiary education, 34.8% (80/230) had secondary education, 23.0% (53/230) had primary education, and 8.7% (20/230) had no formal education. Over half of the participants had an occupation in trading (51.1%, 117/230), followed by civil servants (17.9%, 41/230), self-employed (8.3%, 19/230), farmers (7.0%, 16/230), and others (12.7%, 29/230). A summary of the baseline demographic characteristics is presented in Table 1. The majority presented with advanced-stage (III/IV) disease: 73.0% (168/230). Molecular subtyping based on immunohistochemistry results was available for only 59 of 230 participants (25.7%), as testing was not consistently available across centres. Among those with available subtype data, 76.3% (45/59) were classified as triple-negative breast cancer (Table 2). Given the high proportion of missing subtype data, these results should be interpreted as reflecting the tested subset rather than the full cohort.

### Knowledge of breast cancer risk factors

Knowledge of established breast cancer risk factors was low across all three centres, with a mean correct response rate of 20.5% (SD 14.8%) across 13 established risk factor items (Supplementary Table 1, Supplementary Fig. 1). The most recognized risk factor was a prior history of breast cancer (55.2%, 127/230), whereas recognition of modifiable factors such as physical inactivity (7.4%, 17/230), obesity (9.6%, 22/230), and daily alcohol consumption (19.6%, 45/230) was poor. Fewer than half (43.9%, 101/230) of the patients recognized family history as a risk factor.

The recognition that cultural beliefs and practices are not breast cancer risk factors was low across the cohort, with a mean correct response rate of 42.2% (SD 8.9%) across the six misconception items. The proportions of participants who correctly rejected each item as a risk factor were as follows: exposure to dirty air or water (52.2%, 120/230), wearing a bra at night (49.6%, 114/230), wearing a tight bra (46.3%, 106/230), witchcraft or evil spirits (40.4%, 92/230), putting money in one’s bra (35.4%, 81/230), and keeping a mobile phone in one’s bra (29.1%, 67/230). In the multivariable linear regression, higher risk factor knowledge scores were independently associated with education (*p* = 0.034), SES (*p* < 0.001), age group (*p* = 0.037), and hospital centre (*p* = 0.043) (Supplementary Fig. 2, Supplementary Table 3).

### Knowledge of breast cancer signs and symptoms

Knowledge of breast cancer signs and symptoms was low, with marked variation across centres (Supplementary Table 2, Supplementary Fig. 3). The most recognized symptom was breast lump (63.5%, 146/230), followed by bleeding from the nipple (58.8%, 134/230) and pulling in of the nipple (56.1%, 128/230). Fewer than half of the participants correctly identified changes in breast size (40.9%, 94/230) or changes in breast shape (40.2%, 92/230) as potential warning signs, whereas only 30.9% (71/230) recognized pain in the armpit as a symptom. Compared with OAUTHC and FETHA participants, UITH demonstrated substantially lower symptom knowledge. Notably, “Did not answer” at UITH were also more frequent than at the other two centres. In the multivariable analysis, clinical stage (*p* = 0.021), hospital centre (*p* < 0.001) and tertiary education (*p* = 0.008) remained independently associated with symptom knowledge (Supplementary Fig. 4, Supplementary Table 3).

### Knowledge of hereditary breast cancer

Just over half of the participants (124/223, 55.6%, 95% CI 49.1–61.9%) correctly reported that breast cancer can be hereditary (7 missing responses excluded). In the multivariable logistic regression (adjusted for age, education, hospital centre), education was independently associated with hereditary breast cancer recognition (*p* < 0.001), with progressively higher adjusted odds at each successive educational level compared with no formal education: primary aOR 4.42 (95% CI 1.09–17.93, *p* = 0.038), secondary aOR 8.04 (95% CI 1.96–32.95, *p* = 0.004), and tertiary aOR 16.04 (95% CI 3.87–66.50, *p* < 0.001). Hospital centre was also independently associated with hereditary breast cancer recognition in the adjusted model (*p* = 0.024). Age group was no longer associated after adjustment (*p* = 0.18).

### Awareness of *BRCA1/2*

Awareness of *BRCA1/2* genes was extremely low: only 11 (4.8%, 95% CI 2.7–8.4%) participants reported prior knowledge of these genes. Among this small subgroup, most correctly identified that *BRCA1/2* mutations increase breast cancer risk (90.9%, 10/11), hereditary cancer genes can be transmitted from parents to children (81.8%, 9/11), hereditary breast cancer may require additional treatment (81.8%, 9/11) and that these mutations are also associated with an increased risk of other malignancies (72.7%, 8/11). In contrast, fewer than one-third correctly identified that *BRCA1/2* genes are present in all individuals (27.3%, 3/11). The sources of information included HCPs (54.5%, 6/11) and the internet (45.5%, 5/11).

These 11 participants were predominantly younger than 40 years (72.7%, 8/11), tertiary-educated (72.7%), and 6 (54.5%) were of Igbo ethnicity and recruited from FETHA, and 5 (45.5%) were of Yoruba ethnicity and recruited from OAUTHC; none were recruited from UITH. SES was distributed between low (6/11) and middle (5/11) categories, with no high-SES participants in this subgroup. The clinical stage at diagnosis was available for 10 participants, among whom 40.0% (4/10) had early-stage disease (stages 0-II), 20.0% (2/10) had locally advanced disease (stage III), and 40.0% (4/10) had advanced or metastatic disease (stage IV). Molecular subtype information was available for five participants; of these, 4 had triple-negative breast cancer (Table 3).

### Willingness to undergo *BRCA1/2* testing and family disclosure

Willingness to engage in genetic testing was high across the cohort. Nearly all participants indicated that they would undergo *BRCA1/2* testing if offered (95.7%, 220/230), would inform their siblings if they were diagnosed with hereditary breast cancer (94.8%, 218/230), and would encourage relatives to undergo testing (96.1%, 221/230). Among the 11 participants who were aware of *BRCA1/2*, willingness remained high: 81.8% (9/11) were willing to undergo testing, 81.8% (9/11) were willing to inform their siblings, and 90.9% (10/11) were willing to encourage relatives to test. Given the ceiling effect in these outcomes, no significant associations were observed in univariable analyses of education, SES, age, ethnicity, religion, hospital centre, or cancer stage.

For all participants, disclosure of their diagnosis was predominantly family-centered. Among the 230 participants, the first person informed was most commonly a spouse (98/230, 42.6%), followed by a son or daughter (49/230, 21.3%), a sibling (29/230, 12.6%), a parent (7/230, 3.0%), and a member of the extended social or religious network (9/230, 3.9%). Among the 140 participants who provided a reason for their choice, the most frequently cited motivations were the need for a trusted confidant or advisor (30.0%), reliance on a primary support figure or household decision-maker (20.0%), provision of caregiving (17.9%), and practical reasons such as assistance with transport (16.4%).

## Discussion

In this multicentre study of 230 newly diagnosed patients with breast cancer recruited from three tertiary hospitals in Nigeria, awareness of *BRCA1/2* was extremely low (4.8%), whereas willingness to undergo germline testing was high. The gap between these two findings has direct implications for how hereditary cancer services are designed in resource limited settings where germline testing will be offered for the first time.

General knowledge of established breast cancer risk factors was low across all three centres, the average rate of correct responses was 20.5%, and misconceptions including attributing breast cancer to spiritual or supernatural causes were common. These findings are broadly consistent with prior Nigerian and West African literature, [17–20] and align with beliefs previously documented in both community settings and among HCPs [21, 22]. Fewer than half of all participants recognized family history as a personal risk factor for breast cancer, suggesting that patients in this setting are unlikely to self-identify as candidates for germline testing based on their own perception of family risk, and that the rationale for testing will need to be introduced by clinicians rather than patient knowledge.

Just over half of the participants recognized that breast cancer can be hereditary, despite having little or no knowledge of specific susceptibility genes. The strongest predictor was education, which showed a graded relationship, and each successive level of education was associated with substantially higher odds of recognizing hereditary risk than with those who had no formal education. Although formal education provides a foundational capacity to understand the concept of inherited cancer risk, future consent processes and patient-facing materials will need to be comprehensible across the full educational gradient present in Nigerian patient populations. Importantly, gaps in genetic literacy are not necessarily closed by exposure to counselling. In a recent study from Mexico, PV carriers who had received post-test genetic counselling retained persistent gaps in understanding inheritance patterns and interpretation of results, with education remaining independently associated with better comprehension [23]. Genetic literacy gaps in this setting are therefore unlikely to be resolved by counselling alone, and educational materials and consent pathways must be designed to support all levels of comprehension.

Awareness of *BRCA1/2* was extremely low in our cohort, and lower than previously reported among Nigerian professional women without a personal history of breast cancer [15]. The 11 participants with prior *BRCA1/2* awareness were predominantly younger, tertiary-educated women recruited from FETHA and OAUTHC, with low- and middle-income backgrounds. Understanding among these participants was inconsistent, although most correctly identified that *BRCA1/2* mutations increase breast cancer risk and that hereditary cancer genes can be transmitted from parents to children; however, fewer than one-third recognize that *BRCA1/2* genes are present in all individuals. This subgroup also exhibited high-risk clinical features, including early-onset disease, a high proportion of triple-negative breast cancer among those with available subtype data, and advanced-stage presentation. These features are characteristic of hereditary breast cancer and highlight the potential clinical utility of genetic testing for these patients. HCPs and the internet were the only reported information sources, highlighting the central role of clinicians in communicating genetic risk information. Nigerian pilot studies have demonstrated that targeted education of HCPs can substantially improve genetic knowledge [24, 25], and support the uptake of genetic testing among patients [26]. At the same time, strengthening provider knowledge must be accompanied by attention to practical counselling considerations in resource limited settings. Previously reported challenges from a provider perspective include language limitations in explaining genetic concepts in different languages, difficulty communicating different types of genetic results, and challenges in conveying the lifelong and familial implications of PVs [27]. In diverse sociocultural contexts, providers will be required to translate complex genomic information into culturally and linguistically appropriate counselling to ensure that access to testing is accompanied by meaningful patient understanding.

Willingness to undergo *BRCA1/2* testing was nearly universal in this cohort, despite extremely low baseline awareness of *BRCA1/2*. This intent remained high when confined to the 11 participants with prior awareness of *BRCA1/2*, comparable acceptability has been reported among Nigerian professional women as well as among cancer patients in Nigeria and Cameroon, who were surveyed after receiving structured pre-test genetic counselling [15, 16, 28], together indicating that the acceptability of germline testing is not a fundamental barrier in this setting. However, translating this acceptability into hereditary breast cancer services will require navigating several interrelated challenges including affordability, service infrastructure, and the acceptability and feasibility of risk-reducing interventions.

In literature, the cost of testing remains the most frequently cited barrier for low- and middle-income settings, as out-of-pocket germline testing can cost several times the annual per-capita health expenditure, and is rarely subsidized by public health systems or insurance [29, 30]. Evidence from South West Nigeria indicate that patients were willing to pay below 100 US dollars [16]. However, this may be overstated among patients already struggling to finance their treatment. As the cost of next-generation sequencing continues to fall, the longer-term priority is developing in-country testing capacity to reduce dependence on costly external laboratories and expanding the National Health Insurance Scheme to cover genetic testing [13, 31]. Compounding this is the absence of data describing clinical management of hereditary breast cancer in this setting, which will be needed to ensure that testing is coupled to actionable and locally available treatment pathways. For patients identified as carriers, the value of testing depends on the acceptability and feasibility of risk-reducing surgery including risk-reducing mastectomy and risk-reducing salpingo-oophorectomy [32]. In a survey of healthy, educated Nigerian women, acceptance of bilateral prophylactic mastectomy was low (37.4%), the principal deterrents being anticipated loss of self-esteem, body image and fear of surgery [33]. These findings suggest that attitudes toward risk reducing surgery are predominantly psychosocial and sociocultural, and that interventions to support informed decision-making will need to address these cultural norms alongside genetic education. As these concerns stem largely from the anticipated physical and psychological impact of mastectomy, the availability of reconstruction options may be an important determinant in the acceptability of risk-reducing mastectomy [34].

These findings have direct implications for the design of informed consent, in a population with very limited baseline understanding of hereditary breast cancer, genetic testing and patient willingness to undergo testing cannot assume informed comprehension of what a positive test result entails – including surveillance, risk-reducing surgery, and family-communication decisions that may follow. Even clinical genetics services from Western counterparts have shown that even when significant time is invested in pre-test counselling, patients frequently leave consultations with limited understanding of the test or its implications, and decisions to undergo testing are often shaped by trust in the clinician [35]. Consent processes will therefore need to be designed as collaborative and ongoing, so that patient understanding develops across the clinical journey, rather than a single transfer of information at the time of testing [35]. Further, our data also revealed high overall willingness to inform relatives and to encourage family members to be tested as well. Patterns of disclosure have direct implications for cascade testing, which relies on patients communicating genetic risk information to first degree relatives.

Taken together with previously reported provider readiness [13], our findings indicate that the principal barriers to introducing hereditary cancer services in Nigeria are service availability and patient and community awareness rather than patient or provider acceptability. An education scale-up therefore represents a critical next step in this setting. Direct translation of hereditary cancer education templates developed in higher-income contexts is insufficient. Even within high income settings, the development of patient education materials for Black women has shown that generic content fails to engage target audiences without contextual adaptation in language, imagery, or the specific examples used to convey hereditary risk [36]. Importantly, this adaptation must extend to engage the psychosocial and sociocultural beliefs that shape how hereditary risk and risk-reducing options are understood and accepted in this setting. Future patient-facing educational frameworks for this setting should therefore, in plain language, introduce basic genetic concepts (genes, PVs, and how they are inherited); the distinction between hereditary and sporadic breast cancer, and the family-history features that may indicate hereditary risk (early age at diagnosis, multiple affected relatives, and other malignancies). It should also explain methods of extracting DNA for genetic testing (e.g. blood or saliva), the three possible result categories (pathogenic mutation, no mutation detected, and variants of uncertain significance [VUS]), and how each result may influence cancer treatment decisions, screening recommendations, risk-reduction options, and cascade testing for family members. This mode of context-adapted educational intervention has already been shown to significantly improve knowledge of breast cancer risk, screening, and familial risk among newly diagnosed patients with initially low baseline awareness in North India [37].

A key strength of this study was the inclusion of participants from three tertiary teaching hospitals spanning three Nigerian geopolitical zones. In addition, the questionnaire was administered at the point of diagnosis, capturing baseline patient literacy at the clinical moment at which germline testing would first be introduced. Several limitations should be acknowledged. First, the questionnaire was interviewer-administered in a clinical setting by research staff, this design may introduce social desirability bias. Further, the site-level differences in symptom knowledge we observed should be interpreted in the context of questionnaire administration, as discussed in the results. A further limitation concerns the interpretation of willingness. As most participants had limited genetic literacy at diagnosis, their intent to undergo testing may have been expressed without full comprehension of what testing and result interpretation involves. Prospective studies should therefore capture this nuance before and after a genetics education intervention, as a more reliable estimate of informed willingness. The 49-item questionnaire was not formally validated against pre-existing instruments such as the KnowGene Cancer Genetics Questionnaire [38] and the 27-item Breast Cancer Genetic Counseling Knowledge Questionnaire (BGKQ) [39]; these existing instruments were developed for United States genetic counselling practice and have not been adapted for SSA populations, and future work should address the absence of a validated, context-appropriate instrument for measuring hereditary cancer literacy in this setting. As a next step, qualitative interview data will be examined to provide deeper insight into the emotional, social, and cultural determinants of participant perspectives, and to clarify patterns of communication within families. Future work should also examine the willingness to consider risk-reducing surgical options among patients who meet established criteria for hereditary cancer testing.

In conclusion, our multicentre findings demonstrate that awareness of hereditary breast cancer remains limited among newly diagnosed patients in Nigeria, despite strong interest in *BRCA1/2* testing and willingness to encourage testing among family members. This indicates that high demand for genetic services is not accompanied by sufficient understanding to support informed decision-making. Knowledge of hereditary breast cancer was strongly patterned by education. Culturally tailored education within routine care settings alongside targeted training of HCPs [24, 25] offers a practical approach to improving genetic literacy and supporting willingness among newly diagnosed patients and their asymptomatic first-degree relatives. These implementation approaches are critical as hereditary breast cancer services are being developed in Nigeria and comparable SSA settings.

**Table 1:** Sociodemographic data

	*n* = 230
**Hospital Centre**	
OAUTHC, Ile-Ife	99 (43.0)
UITH, Ilorin	74 (32.2)
FETHA, Abakaliki, Ebonyi	57 (24.8)
**Sex**	
Female	228 (99.1)
Male	1 (0.4)
Not reported	1 (0.4)
**Age**	
Mean (sd)	48.3 (11.7)
Median (Min, Max)	47 (23, 87)
**Occupation**	
Trading	117 (51.1)
Civil Servant	41 (17.9)
Other^1^	29 (12.7)
Self Employed	19 (8.3)
Farmer	16 (7.0)
Pensioner	4 (1.7)
Unemployed	3 (1.3)
Not reported	1
**Socio economic status**	
Low	153 (66.5)
Middle	71 (30.9)
High	5 (2.2)
Not reported	1 (0.4)
**Ethnicity**	
Yoruba	164 (71.3)
Hausa	2 (0.9)
Igbo	54 (23.5)
Others^2^	10 (4.3)
**Educational Status**	
None	20 (8.7)
Primary	53 (23.0)
Secondary	80 (34.8)
Tertiary	76 (33.0)
Not reported	1 (0.4)
**Religion**	
Christianity	159 (69.1)
Islam	70 (30.4)
Traditional	1 (0.4)

"Other" occupations (n = 29) included teaching (17.2%), business and hairdressing (10.3% each), fashion design, tailoring, retiree, attendant (6.9% each), and single mentions (3.4% each) such as banker, housewife, makeup artist, prophetess, seamstress, student, system analyst

"Other" ethnicities (n = 10) included Bette, Nupe, Boki, Cross River, Obudu, Urhobo, Yahe, and Yala

**Table 2:** Clinical Characteristics

**Stage**	*n = 230*	
I	6 (2.6)	
II	48 (20.9)	
III	119 (51.7)	
IV	49 (21.3)	
Not reported	8 (3.5)	
**Molecular Subtype**		*n* = 59
Luminal A (ER + and/or PR +, HER-2 -)		12 (20.3)
Luminal B (ER + and/or PR +, HER-2 +)		1 (1.7)
HER-2 (ER and PR -, HER-2 +)		1 (1.7)
Triple negative (ER/PR -, HER-2 -)		45 (76.3)
Not reported		171 (74.4)

**Table 3:** Sociodemographic and clinical profiles of participants with prior awareness of *BRCA1/2*

**Hospital Centre**	*n* = 11
OAUTHC, Ile-Ife	5 (45.5)
UITH, Ilorin	0 (0.0)
FETHA, Abakaliki, Ebonyi	6 (54.5)
**Age group**	
0–39	8 (72.7)
40–64	2 (18.2)
65 and above	1 (9.1)
**Socio economic status**	
Low	6 (54.5)
Middle	5 (45.5)
High	0 (0.0)
**Ethnicity**	
Other	1 (9.1)
Yoruba	4 (36.4)
Hausa	0 (0.0)
Igbo	6 (54.5)
**Educational Status**	
None/Prefer not to disclose	0 (0.0)
Primary	0 (0.0)
Secondary	3 (27.3)
Tertiary	8 (72.7)
**Religion rev**	
Christianity	11 (100.0)
**Stage grouping rev**	
Early Breast Cancer (0,I, II)	4 (40.0)
Locally Advanced (III)	2 (20.0)
Advanced/Metastasis (IV)	4 (40.0)
Not reported	1
**Molecular Subtype rev**	
Luminal A (ER + and/or PR +, HER-2 -)	1 (20.0)
Luminal B (ER + and/or PR +, HER-2 +)	0 (0.0)
HER-2 (ER and PR -, HER-2 +)	0 (0.0)
Triple Negative (ER/PR -, HER-2 -)	4 (80.0)
Not reported	6

## Supplementary Information

Below is the link to the electronic supplementary material.

Supplementary Material 1

Supplementary Material 2

Supplementary Material 3

Supplementary Material 4

Supplementary Material 5

Supplementary Material 6

Supplementary Material 7

## Data Availability

All data supporting the findings of this study are available within the paper and its Supplementary Information.
