# Supplementary material for: Awareness of hereditary breast cancer and readiness for genetic testing among newly diagnosed patients with breast cancer in Nigeria: a multicentre study
Source: Hered Cancer Clin Pract. 2026 Jul 27;24:22. doi: 10.1186/s13053-026-00349-8 (PMC13688898; doi:10.1186/s13053-026-00349-8)
Supplement: Supplementary file 2 [file 13053_2026_349_MOESM2_ESM.docx]

**Figure 2: Distribution of knowledge scores for breast cancer risk factors by**

**sociodemographic characteristics and cancer stage**
