# Supplementary material for: Awareness of hereditary breast cancer and readiness for genetic testing among newly diagnosed patients with breast cancer in Nigeria: a multicentre study
Source: Hered Cancer Clin Pract. 2026 Jul 27;24:22. doi: 10.1186/s13053-026-00349-8 (PMC13688898; doi:10.1186/s13053-026-00349-8)
Supplement: Supplementary file 3 [file 13053_2026_349_MOESM3_ESM.docx]

**Figure 3: Knowledge of breast cancer nipple-related, breast change, and systemic signs and symptoms by tertiary hospital**
