# Supplementary material for: Awareness of hereditary breast cancer and readiness for genetic testing among newly diagnosed patients with breast cancer in Nigeria: a multicentre study
Source: Hered Cancer Clin Pract. 2026 Jul 27;24:22. doi: 10.1186/s13053-026-00349-8 (PMC13688898; doi:10.1186/s13053-026-00349-8)
Supplement: Supplementary file 5 [file 13053_2026_349_MOESM5_ESM.docx]

**Supplementary Table 1: Knowledge of breast cancer risk factors by tertiary hospital**

|  | Risk Factor | Overall  n (%) correct | OAUTHC  n (%) correct | UITH  n (%) correct | FETHA  n (%) correct | |
| --- | --- | --- | --- | --- | --- | --- |
| Established Risk Factors | Having had breast cancer previously | **127 (55.2)** | 55 (55.6) | 49 (66.2) | | 23 (40.4) |
|  | Drinking more than 1 bottle or 1 glass of alcohol per day | **45 (19.6)** | 9 (9.1) | 24 (32.4) | | 12 (21.1) |
|  | Not breastfeeding | **48 (21.0)** | 11 (11.1) | 21 (28.4) | | 16 (28.6) |
|  | Using hormone replacement therapy | **49 (21.4)** | 4 (4.1) | 27 (36.5) | | 18 (31.6) |
|  | Using hormonal family planning methods (e.g birth control pill, injectable contraceptives and implants) | **68 (29.6)** | 7 (7.1) | 36 (48.6) | | 25 (43.9) |
|  | Being overweight | **22 (9.6)** | 11 (11.1) | 3 (4.1) | | 8 (14.0) |
|  | Early menarche <11 years | **20 (8.7)** | 7 (7.1) | 2 (2.7) | | 11 (19.3) |
|  | Physical inactivity | **17 (7.4)** | 7 (7.1) | 3 (4.1) | | 7 (12.3) |
|  | Ageing/growing old | **49 (21.3)** | 29 (29.3) | 6 (8.1) | | 14 (24.6) |
|  | Nulliparity | **21 (9.1)** | 9 (9.1) | 2 (2.7) | | 10 (17.5) |
|  | Late menopause (>55) | **25 (10.9)** | 10 (10.1) | 1 (1.4) | | 14 (24.6) |
|  | Family history of breast cancer | **101 (43.9)** | 36 (36.4) | 39 (52.7) | | 26 (45.6) |
|  | First child after age 30 | **20 (8.7)** | 12 (12.1) | 2 (2.7) | | 6 (10.7) |
|  | **Mean (sd) % Correct Across Items** | **20.5% (14.8%)** | **16.1% (15.1%)** | **22.4% (23.0%)** | | **25.7% (11.8%)** |
| Cultural Beliefs and Misconceptions | Witchcraft/evil spirits | **92 (40.4)** | 27 (27.6) | 49 (67.1) | | 16 (28.1) |
|  | Wearing a tight bra | **106 (46.3)** | 63 (64.3) | 25 (33.8) | | 18 (31.6) |
|  | Wearing bra at night | **114 (49.6)** | 66 (66.7) | 30 (40.5) | | 18 (31.6) |
|  | Putting money in bra | **81 (35.4)** | 50 (50.5) | 18 (24.7) | | 13 (22.8) |
|  | Keeping phone in bra | **67 (29.1)** | 39 (39.4) | 17 (23.0) | | 11 (19.3) |
|  | Exposure to dirty air/water | **120 (52.2)** | 74 (74.7) | 15 (20.3) | | 31 (54.4) |
|  | **Mean (sd) % Across Items** | **42.2% (8.9%)** | **53.9% (18.0%)** | **34.9% (17.5%)** | | **31.3% (12.3%)** |
|  |  |  |  |  | |  |
