# Supplementary material for: Awareness of hereditary breast cancer and readiness for genetic testing among newly diagnosed patients with breast cancer in Nigeria: a multicentre study
Source: Hered Cancer Clin Pract. 2026 Jul 27;24:22. doi: 10.1186/s13053-026-00349-8 (PMC13688898; doi:10.1186/s13053-026-00349-8)
Supplement: Supplementary file 6 [file 13053_2026_349_MOESM6_ESM.docx]

**Supplementary Table 2: Knowledge of breast cancer signs and symptoms by tertiary hospital**

| Symptom | Overall n (%) | OAUTHC  n (%) correct | UITH  n (%) correct | FETHA  n (%) correct |
| --- | --- | --- | --- | --- |
| Change in nipple position | 121 (52.8%) | 78 (78.8%) | 6 (8.2%) | 37 (64.9%) |
| Pulling in of the nipple | 128 (56.1%) | 85 (86.7%) | 1 (1.4%) | 42 (73.7%) |
| Change in nipple size (not pregnant/breastfeeding) | 99 (43.2%) | 58 (58.6%) | 3 (4.1%) | 38 (66.7%) |
| Change in nipple shape (not pregnant/breastfeeding) | 92 (40.0%) | 53 (53.5%) | 2 (2.7%) | 37 (64.9%) |
| Nipple rash | 79 (34.5%) | 61 (62.2%) | 0 (0.0%) | 18 (31.6%) |
| Nipple discharge (not pregnant/breastfeeding) | 108 (47.0%) | 67 (67.7%) | 14 (18.9%) | 27 (47.4%) |
| Bleeding from the nipple | 134 (58.8%) | 69 (71.1%) | 29 (39.2%) | 36 (63.2%) |
| Fever | 164 (71.6%) | 84 (85.7%) | 47 (63.5%) | 33 (57.9%) |
| Pain in one/both breasts (not pregnant/menstruating) | 110 (48.0%) | 71 (71.7%) | 4 (5.5%) | 35 (61.4%) |
| Lump/thickening in the breast | 146 (63.5%) | 88 (88.9%) | 14 (18.9%) | 44 (77.2%) |
| Change in breast skin color (not pregnant/breastfeeding) | 101 (43.9%) | 60 (60.6%) | 2 (2.7%) | 39 (68.4%) |
| Puckering or dimpling of breast skin | 125 (54.8%) | 87 (88.8%) | 0 (0.0%) | 38 (66.7%) |
| Change in breast size (not pregnant/breastfeeding) | 94 (40.9%) | 52 (52.5%) | 3 (4.1%) | 39 (68.4%) |
| Change in breast shape (not pregnant/breastfeeding) | 92 (40.2%) | 50 (51.0%) | 3 (4.1%) | 39 (68.4%) |
| Lump/thickening under the armpit | 109 (47.4%) | 64 (64.6%) | 5 (6.8%) | 40 (70.2%) |
| Pain in the armpit | 71 (30.9%) | 48 (48.5%) | 6 (8.1%) | 17 (29.8%) |
| Mean (sd) % Across Items | 48.4% (10.8%) | 68.2% (14.1%) | 11.8% (17.0%) | 61.3% (13.7%) |
