# Supplementary material for: Awareness of hereditary breast cancer and readiness for genetic testing among newly diagnosed patients with breast cancer in Nigeria: a multicentre study
Source: Hered Cancer Clin Pract. 2026 Jul 27;24:22. doi: 10.1186/s13053-026-00349-8 (PMC13688898; doi:10.1186/s13053-026-00349-8)
Supplement: Supplementary file 7 [file 13053_2026_349_MOESM7_ESM.docx]

**Supplementary Table 3. Univariable and multivariable linear regression of patient characteristics associated with breast cancer knowledge scores**

**Panel A. Knowledge of breast cancer risk factors**

| **Characteristic** | **Unadjusted Estimate(95%CI)** | **p** | **Adjusted Estimate(95%CI)** | **p (adj)** |
| --- | --- | --- | --- | --- |
| **Hospital Centre** |  | 0.81 |  | **0.043** |
| OAUTHC, Ile-Ife | Reference |  | Reference |  |
| UITH, Ilorin | -1.72 (-7.00, 3.56) | 0.52 | -8.39 (-15.29, -1.50) | **0.017** |
| FETHA, Abakaliki, Ebonyi | -0.54 (-6.25, 5.17) | 0.85 | -1.38 (-7.16, 4.40) | 0.64 |
| **Age group** |  | **0.014** |  | **0.037** |
| 0-39 | Reference |  | Reference |  |
| 40-64 | -6.70 (-11.97, -1.43) | **0.013** | -6.63 (-11.76, -1.50) | **0.012** |
| 65 and above | -10.86 (-19.66, -2.07) | **0.016** | -7.44 (-16.07, 1.19) | 0.091 |
| **Socio economic status** |  | **<0.001** |  | **<0.001** |
| Low | Reference |  | Reference |  |
| Middle | 10.58 (5.85, 15.31) | **<0.001** | 13.98 (8.13, 19.83) | **<0.001** |
| High | 14.23 (-0.74, 29.20) | 0.062 | 19.01 (3.01, 35.01) | **0.020** |
| **Ethnicity** |  | 0.28 |  |  |
| Other | Reference |  |  |  |
| Yoruba | -10.86 (-21.99, 0.27) | 0.056 |  |  |
| Hausa | -13.68 (-40.16, 12.79) | 0.31 |  |  |
| Igbo | -9.49 (-21.26, 2.27) | 0.11 |  |  |
| **Educational Status** |  | **<0.001** |  | **0.034** |
| None/Prefer not to disclose | Reference |  | Reference |  |
| Primary | 10.71 (2.43, 18.99) | **0.011** | 7.94 (-0.34, 16.22) | 0.060 |
| Secondary | 16.19 (8.32, 24.07) | **<0.001** | 10.79 (2.23, 19.34) | **0.014** |
| Tertiary | 21.63 (13.71, 29.54) | **<0.001** | 13.44 (4.41, 22.47) | **0.004** |
| **Religion** |  | 0.095 |  |  |
| Christianity | Reference |  |  |  |
| Islam | -4.17 (-9.07, 0.73) |  |  |  |
| **Stage grouping** |  | 0.62 |  |  |
| Early Breast Cancer (0,I,II) | Reference |  |  |  |
| Locally Advanced (III) | -1.79 (-7.42, 3.84) | 0.53 |  |  |
| Advanced/Metastasis (IV) | 0.86 (-5.90, 7.63) | 0.80 |  |  |

**Panel B. Knowledge of breast cancer signs and symptoms**

|  | **Unadjusted Estimate(95%CI)** | **p** | **Adjusted Estimate(95%CI)** | **p (adj)** |
| --- | --- | --- | --- | --- |
| **Hospital Centre** |  | **<0.001** |  | **<0.001** |
| OAUTHC, Ile-Ife | Reference |  | Reference |  |
| UITH, Ilorin | -56.13 (-63.35, -48.90) | **<0.001** | -52.28 (-63.80, -40.76) | **<0.001** |
| FETHA, Abakaliki, Ebonyi | -6.57 (-14.39, 1.25) | 0.099 | -3.27 (-13.58, 7.03) | 0.53 |
| **Age group** |  | 0.23 |  |  |
| 0-39 | Reference |  |  |  |
| 40-64 | -6.13 (-16.78, 4.52) | 0.26 |  |  |
| 65 and above | -14.82 (-32.60, 2.95) | 0.10 |  |  |
| **Socio economic status** |  | **<0.001** |  | 0.47 |
| Low | Reference |  | Reference |  |
| Middle | -24.05 (-33.30, -14.79) | **<0.001** | 4.64 (-4.64, 13.92) | 0.33 |
| High | -41.62 (-70.90, -12.33) | **0.006** | -4.00 (-28.11, 20.11) | 0.74 |
| **Ethnicity** |  | **0.003** |  |  |
| Other | Reference |  |  |  |
| Yoruba | -14.64 (-36.39, 7.11) | 0.19 |  |  |
| Hausa | -39.37 (-91.10, 12.35) | 0.13 |  |  |
| Igbo | 3.56 (-19.42, 26.55) | 0.76 |  |  |
| **Educational Status** |  | **<0.001** |  | **0.043** |
| None/Prefer not to disclose | Reference |  | Reference |  |
| Primary | 8.78 (-7.97, 25.53) | 0.30 | 10.05 (-2.90, 22.99) | 0.13 |
| Secondary | 27.39 (11.46, 43.32) | **<0.001** | 10.33 (-2.45, 23.10) | 0.11 |
| Tertiary | 32.50 (16.48, 48.51) | **<0.001** | 18.33 (4.94, 31.72) | **0.008** |
| **Religion** |  | **<0.001** |  |  |
| Christianity | Reference |  |  |  |
| Islam | -35.92 (-44.55, -27.29) |  |  |  |
| **Stage grouping** |  | **<0.001** |  | **0.021** |
| Early Breast Cancer (0,I,II) | Reference |  | Reference |  |
| Locally Advanced (III) | -21.64 (-32.27, -11.02) | **<0.001** | -1.63 (-9.98, 6.71) | 0.70 |
| Advanced/Metastasis (IV) | 2.97 (-9.80, 15.75) | 0.65 | 10.59 (0.43, 20.75) | **0.041** |

CI, confidence interval. Reference categories are indicated as “Reference.”
