# Supplementary material for: Awareness of hereditary breast cancer and readiness for genetic testing among newly diagnosed patients with breast cancer in Nigeria: a multicentre study
Source: Hered Cancer Clin Pract. 2026 Jul 27;24:22. doi: 10.1186/s13053-026-00349-8 (PMC13688898; doi:10.1186/s13053-026-00349-8)
Supplement: Supplementary file 8 [file 13053_2026_349_MOESM1_ESM.docx]

**Figure 1: Knowledge of breast cancer risk factors by tertiary hospital**
